# Role of Starter Cultures on the Safety of Fermented Meat Products

**DOI:** 10.3389/fmicb.2019.00853

**Published:** 2019-04-26

**Authors:** Marta Laranjo, Maria Eduarda Potes, Miguel Elias

**Affiliations:** ^1^ICAAM-Instituto de Ciências Agrárias e Ambientais Mediterrânicas, Universidade de Évora, Pólo da Mitra, Évora, Portugal; ^2^Departamento de Medicina Veterinária, Escola de Ciências e Tecnologia, Universidade de Évora, Pólo da Mitra, Évora, Portugal; ^3^Departamento de Fitotecnia, Escola de Ciências e Tecnologia, Universidade de Évora, Pólo da Mitra, Évora, Portugal

**Keywords:** starters, biogenic amines, polyciclic aromatic hydrocarbons (PAHs), lactic acid bacteria, food pathogens, spoilage microbiota, bacteriocins, mycotoxins

## Abstract

Starters are microbial cultures used to promote and conduct the fermentation of meat products. Bacteria, particularly lactic acid bacteria (LAB) and coagulase-negative staphylococci (CNS), as well as yeasts and molds, may be used as starters. They can increase the safety of fermented meat products by means of rapid matrix acidification or due to the production of antimicrobial substances, such as bacteriocins. Besides, starters may help to standardize product properties and shorten ripening times. Safety of fermented meat products may be jeopardized by microbiological, namely foodborne pathogens (*Salmonella* spp., *Listeria* spp., etc), and chemical hazards, particularly biogenic amines, nitrosamines, polycyclic aromatic hydrocarbons (PAH), and mycotoxins. Biogenic amines (BA) are potentially unsafe nitrogenous compounds that result from the decarboxylation of some amino acids. Some microorganisms may be responsible for their formation. Starters can cause a fast pH decrease, inhibiting the development of microorganisms with amino acid decarboxylative ability, thus preventing the accumulation of BA in fermented meat products. Besides, starters can compete with the autochthonous, non-starter microbiota throughout ripening and storage, thus reducing BA production. Some strains of *Lactobacillus sakei* and *Lactobacillus plantarum* have been shown to reduce the formation/accumulation of BA. On the other hand, *Staphylococcus xylosus* and *Debaryomyces hansenii* strains have been reported to degrade BA in food. PAH are organic compounds containing multiple aromatic rings and produced by the incomplete combustion of organic matter, such as the wood used for smoking meat. Mixed starters containing *Lactobacillus* spp., Gram-positive catalase-positive cocci and yeasts have been used in the manufacturing of traditional meat sausages. However, the effect of starters on reducing the accumulation of PAH is poorly understood. Starters may also be engaged in competitive exclusion, outcompeting the spoiling or deteriorating autochthonous microbiota. For example, *Pediococcus acidilactici* has been shown to inhibit *Listeria monocytogenes* in meat products. Additionally, the role of molds, such as *Penicillium nalgiovense*, in the competitive exclusion of undesired filamentous fungi, has also been demonstrated. Most of these undesired fungi produce mycotoxins, secondary metabolites capable of causing disease. The current review addresses the role of starters on the microbiological and chemical safety of fermented meat products.

## Starter Cultures and How to Select Them

Starter cultures or starters are individual or mixed microbial cultures used in known concentrations to promote and conduct fermentation in meat products. Bacteria, particularly lactic acid bacteria (LAB) and coagulase-negative staphylococci (CNS), as well as yeasts and molds, may be used as starters, thus contributing to increase the safety of fermented meat products. Besides starters may help to standardize product properties and shorten ripening times of fermented meat products.

Starter cultures, which are considered as GRAS (Generally Regarded As Safe) by the US Food and Drug Administration (FDA), are able to inhibit the growth of undesirable microbiota, namely pathogenic and spoilage microorganisms (Holzapfel et al., 2003; Young and O’sullivan, 2011; Fraqueza et al., 2016).

Selection criteria for starter cultures should take into account the raw material, the properties of the strain(s), food safety requirements, and quality attributes (Holzapfel et al., 2003).

At present, the use of starter cultures in the manufacture of meat products has been subject of special attention. They are used in traditional products all over the world, as from Turkey, Croatia, Romania, Greece, Italy, Spain, Portugal, Thailand, and China (Hampikyan and Ugur, 2007; Luxananil et al., 2009; Baka et al., 2011; Wang et al., 2013; Ciuciu Simion et al., 2014; Elias et al., 2014; Marusic et al., 2014). The application of these cultures is an important and sustainable method for the conservation of some food products, with recognized technological advantages.

Different species and strains of microorganisms are used as starter cultures constraining the growth of pathogenic microorganisms and suppressing the development of spoilage microorganisms (Šuškovič et al., 2010; Wang et al., 2013). Depending on technological requirements and consumer preferences, different strains are used in different products (Krockel, 2013).

In meat products, the most widely used starter cultures are LAB (Gram-positive, catalase-negative cocci or bacilli), Gram-positive, catalase-positive cocci, mainly CNS, and Micrococcaceae, molds or yeasts (Laranjo et al., 2017a), whose metabolism produces several compounds with antimicrobial action. These starter microorganisms may be used as single or mixed cultures.

LAB normally used as starters in fermented meat products are usually facultative anaerobes and belong mainly to the genera *Lactobacillus*, *Leuconostoc*, *Pediococcus*, *Lactococcus*, and *Enterococcus* (Fraqueza et al., 2016).

Among CNS, the species most used in the fermentation of meat products are the facultative anaerobes *Staphylococcus carnosus* and *S. xylosus* (Stavropoulou et al., 2018). Within the family Micrococcaceae, *Kocuria* spp., which are aerobes, have been mostly used in the fermentation of sausages (Cocconcelli and Fontana, 2015).

The most common yeasts used as meat starters are *Debaryomyces* spp. and *Candida* spp. that can exhibit an aerobic or a facultatively anaerobic metabolism (Laranjo et al., 2017a).

Both bacterial and yeast starters are inoculated in meat batters (Laranjo et al., 2017a).

Molds starters, as strict aerobes, are surface inoculated and belong mainly to the species *Penicillium nalgiovense* and *P. gladioli* (Berni, 2015; Laranjo et al., 2017a).

## Microbiological Hazards in Fermented Meat Products

The main microbiological hazards that may occur in meat products are the foodborne pathogens *Salmonella* spp., *Campylobacter* spp., *L. monocytogenes*, verocytotoxigenic *Escherichia coli* (VTEC), *Yersinia enterocolitica* and *Yersinia pseudotuberculosis*, as well as the toxins of *Staphylococcus aureus*, *Clostridium perfringens*, and *Clostridium botulinum* (EFSA, 2015).

Several factors will influence the protective ability of starter cultures, such as initial level of contamination, nature of the contaminant species, fermentation time, and storage conditions. For example, if the initial contamination level is high, the use of a starter culture cannot improve the quality of the food product. However, a starter culture has the ability to delay the onset of further contamination, extending the shelf-life of a food product (Young and O’sullivan, 2011).

LAB are the starter cultures mostly involved in preventing or controlling microbiological hazards.

### Effect of LAB Starters

One of the aims of the use of starter cultures is to accelerate the production of lactic acid from the fermentation of sugars. The antimicrobial properties of lactic acid result from the establishment of unfavorable conditions that reduce the growth rate of undesirable microorganisms (Krockel, 2013;Bassi et al., 2015).

Other substances can be produced, such as acetic and propionic acids, ethanol, hydrogen peroxide, reuterin, antimicrobial peptides, and bacteriocins (Caplice and Fitzgerald, 1999; Galvez et al., 2008; Young and O’sullivan, 2011; Reis et al., 2012). These products must be effective against spoilage microorganisms, such as *Pseudomonas* spp., *Clostridium tyrobutyricum*, *Brochothrix thermosphacta*, and also can control the growth of enterobacteria, *E. coli*, *Y. enterocolitica*, *L. monocytogenes*, *C. perfringens* (Baka et al., 2011; Casquete et al., 2012; Cenci-Goga et al., 2012; Pragalaki et al., 2013; Gänzle, 2015; Di Gioia et al., 2016; Laranjo et al., 2017a).

*In vitro* inhibitory capacity of some strains of *Lb. sakei* on *Salmonella* spp., *L. monocytogenes* and *S. aureus* (Diez and Patarata, 2013) and *L. monocytogenes* and *E. coli* O157:H7 (Pragalaki et al., 2013) was proved. Only two strains of *Lb. sakei* were able to inhibit *Salmonella* spp. Interestingly, the ATCC 15521 *Lb. sakei* strain used was not harmful to any pathogenic species tested by Diez and Patarata (2013). Counts of *L. monocytogenes* decreased at days 7 and 15 (Diez and Patarata, 2013; Pragalaki et al., 2013) and *E. coli* O157:H7 decreased sharply in the first 4 days of ripening but both maintained a level of survival of 2.0 log cfu/g after 26 days (Pragalaki et al., 2013).

In *in vitro* experiments, inhibitory capacity of *Lb. plantarum* and *Lactobacillus delbrueckii* against *C. perfringens* and *Clostridium* spp. was demonstrated too (Di Gioia et al., 2016).

The use of *Lb. sakei* strains, *Lb. plantarum* and *Lb. curvatus* separately, in low acid fermented sausage resulted in a marked decrease of enterobacteria (3.5 log cfu/g until less than 1.0 log cfu/g) in 16 days (Baka et al., 2011).

In meat products, Casquete et al. (2012), using a starter culture made up of autochthonous strains of *P. acidilactici* and *Staphylococcus vitulinus*, demonstrated the inhibitory effect on enterobacteria and coliforms in “Salchichón,” a traditional Iberian dry-sausage. Growth inhibition of coliforms and presumed *S. aureus* was also detected in salami inoculated with a starter culture made up of two strains of *Lactococcus lactis* ssp. *lactis* plus *Lactococcus casei* ssp. *casei* (Cenci-Goga et al., 2012). These authors observed the same effect on *Salmonella* spp., *Listeria* spp. and *S. aureus* and attributed it to other inhibitory compounds than acids, since the pH values of the inoculated and control products were very similar.

In pork ground meat for fermented salami preparation, *Lb. plantarum* inoculated at a concentration of 9.0 log cfu/g, inhibited the growth of artificially inoculated (4.0 log cfu/g) *C. perfringens* and *Clostridium spp.* in 2.0 log cfu/g and 1.5 log cfu/g, respectively, after 9 days of fermentation (Di Gioia et al., 2016).

On the other hand, in Dacia, the Romanian traditional dry-sausage, the decrease of Gram-negative microorganisms, namely enterobacteria, throughout the ripening period is explained by the low pH value, due to the inclusion of *Lactobacillus acidophilus* (at a concentration of 8.0 log cfu/g) on the starter culture (Ciuciu Simion et al., 2014).

In sausages inoculated with a 2% *Lb. sakei* suspension (6.0 log cfu/mL), pH values reached 4.52 on 15th day of ripening. This revealed that the acidification occurring in the inoculated sausages is responsible for the inhibition of enterobacteria and *E. coli* until the end of the ripening period although these microorganisms are detected in sausages spontaneously fermented (Wang et al., 2013).

In North European cured raw hams, the LAB *Tetragenococcus halophilus* was used as starter culture combined with *Staphylococcus equorum*, and the result was a 3–5 log reduction in the *S. aureus* counts, together with an improvement in color (Schlafmann et al., 2002).

#### Antimicrobial Effects of Organic Acids

Organic acids, such as lactic, acetic, formic, propionic, and butyric acids, are known to be effective against Gram-positive and Gram-negative bacteria, as well as yeasts.

The antimicrobial effects of organic acids may be played either by the action of undissociated molecules of the organic acids or by the reduction of pH (Pragalaki et al., 2013).

Organic acids produced during fermentation by LAB, like acetic and lactic acid, act by diffusion of the undissociated form of the molecule across the cell membrane. Inside the cell, the molecule dissociates, the pH decreases, and the proton-motive force dissipates, disrupting transport systems and causing the cell destruction (Šuškovič et al., 2010; Pragalaki et al., 2013).

Acidity can also play an additional role on the control of undesirable microorganisms potentiating the effect of other antimicrobial agents. Bactericidal effects of nitrates and nitrites as well as its metabolic intermediates like nitric oxide (NO), nitrogen dioxide (NO_2_), and nitrous oxide (N_2_O) are recognized. These compounds are produced faster in low pH (Wang et al., 2013). Thus, the presence of strains with high acidifying activity can contribute to the improvement of food safety, associated to a decrease in the use of nitrates and nitrites.

#### Antimicrobial Effects of Other Chemical Compounds

In addition the production of lactic acid, some LAB strains are able to produce several other antimicrobial compounds, namely hydrogen peroxide, ethanol, carbon dioxide, and reuterin, among others (Šuškovič et al., 2010; Reis et al., 2012; Mu et al., 2018).

Hydrogen peroxide (H_2_O_2_) is a compound produced by LAB in the presence of oxygen by oxidases like pyruvate oxidases, lactate oxidases, NADH oxidases and flavoproteins reductases in anaerobiosis (Pragalaki et al., 2013; Hertzberger et al., 2014). Its deleterious effects lie on the oxidizing effect of sulfhydryl groups of enzymes and the peroxidation of membrane lipids is the main cause of destruction of microbes (Šuškovič et al., 2010; Pragalaki et al., 2013). Some free radicals that can damage bacterial DNA, such as superoxide (O^2−^) and hydroxyl (OH^−^) may also be produced from H_2_O_2_ (Ammor et al., 2006; Pragalaki et al., 2013).

As suggested by Pragalaki et al. (2013), *L. monocytogenes* and *E. coli* O157:H7 may be inactivated by hydrogen peroxide produced by two autochthonous *Lb. sakei* starter cultures.

Lactic acid bacteria also produce ethanol, which as other volatiles contributes to the typical flavor of some fermented products (Leroy and De Vuyst, 2004; Reis et al., 2012).

Carbon dioxide is a by-product from the fermentation of sugars by heterofermentative LAB. It plays an important role in food preservation replacing the aerobic atmosphere by an anaerobic environment. Its antifungal activity is due to the accumulation in the membrane, compromising its permeability, owing to the inhibition of enzymatic decarboxylations (Šuškovič et al., 2010).

Reuterin (3-hydroxypropionaldehyde) is a well-known broad-range antimicrobial compound produced by *Lactobacillus reuteri* under anaerobic fermentation (Mu et al., 2018). Reuterin may be converted into different compounds, and thus it has been difficult to determine the mechanism by which reuterin exerts its antimicrobial effect (Schaefer et al., 2010). Reuterin is spontaneously converted in acrolein, which is a cytotoxic electrophile, but reuterin and not acrolein is responsible for the antimicrobial action (Schaefer et al., 2010).

The use of reuterin as a possible additive to prevent food spoilage and pathogen growth in different food matrices has been studied (Arqués et al., 2004; Arqués et al., 2008; Montiel et al., 2014). Reuterin was also shown to be effective in reducing the viable cells of *E. coli* O157:H7 and *L. monocytogenes* in pork meat during a 1 week storage period (El-Ziney et al., 1999).

Finally, it has been shown that certain LAB possess a nitrite reductase enzyme system that reduces, under anaerobic conditions, nitrite used as preservative agent in some meat products, suggesting that LAB contribute to the depletion of nitrite in many foods (Wang et al., 2013). This is a relevant fact for food safety considering the recommendations of EFSA (Mortensen et al., 2017a,b) for reducing the utilization of nitrates and nitrites in food preservation.

The presence of nitrate reductase and heme-independent nitrite reductase, able to convert nitrite to NO, NO_2_, and N_2_O has also been described in *Lb. sakei* (Wang et al., 2013). In fact, nitrite concentration was significantly lower in fermented sausages inoculated with *Lb. sakei* than in control sausages, probably due to its nitrite reductase, which is responsible for nitrite depletion (Wang et al., 2013).

This is especially relevant for the control of *L. monocytogenes* and *C. botulinum* that have low concentrations of the enzymes involved in nitrite metabolism (Cammack et al., 1999).

#### Bacteriocins and Other Antimicrobial Peptides

Among the different antimicrobial compounds produced by LAB, bacteriocins have been subject of attention lately. They may be considered an alternative type of antimicrobial agents (Cotter et al., 2005; Hassan et al., 2012; Collins et al., 2017; Mokoena, 2017). They constitute a group of peptides with bactericidal or bacteriostatic activity against species closely related to the producer as some food spoilage and food poisoning Gram-positive bacteria like *Bacillus* spp., *Clostridium* spp., *Staphylococcus* spp., and *Listeria* spp. They are ribosomally synthesized and released extracellularly (Keşka et al., 2017). Examples of bacteriocins are nisin, pediocin, sakacin, curvacin, plantaricin, and bacteriolysins, such as enterolysin A and lysostaphin. They are effective in the control of several species of pathogens including *L. monocytogenes*, *S. aureus, Campylobacter* spp., *E. coli*, *C. perfringens*, and *Bacillus cereus* (Laranjo et al., 2017a).

Several systems have been used to classify bacteriocins with criteria such as structure or antimicrobial action (Fraqueza et al., 2016). The classification of Cotter et al. (2005) is at present generally accepted and classifies bacteriocins into four classes: class I includes the lanthionine-containing bacteriocins/lantibiotics; class II comprises the non-lanthionine-containing bacteriocins; class III are the bacteriolysins or non-bacteriocin lytic proteins, which are nowadays no longer formally considered as bacteriocins; and class IV includes bacteriocins with non-proteinaceous moieties, but no members of this class have been found.

Regarding their range of antimicrobial action, bacteriocins may be divided into three groups: (1) those with a narrow range of antagonist activity, against strains within the same species, or species within the same genus; (2) those with antimicrobial activity against other bacterial species, including the pathogens *L. monocytogenes*, *S. aureus*, *C. perfringens* or *C. botulinum*; and (3) those exhibiting a broad spectrum of inhibiting activity, including Gram-positive and Gram-negative bacteria, and even fungi (Keşka et al., 2017).

However, some authors argue that they are not active against Gram-negative bacteria because of their protective lipopolysaccharide layer which prevents the penetration of antimicrobials, including also bacteriocins (Keşka et al., 2017).

Bacteriocins produced by different LAB species and their target microorganisms are listed in Table 1.

**Table 1:** Types and examples of bacteriocins produced by LAB isolated from meat products.

Bacteriocin	Bacteriocin producing species	Sensitive microorganisms	References
Curvacin	*Lactobacillus curvatus*	*Listeria monocytogenes Staphylococcus aureus Brochothrix thermosphacta Escherichia coli Pseudomonas* spp.	Dicks et al., 2004; Cocolin and Rantsiou, 2007
Nisin	*Lactococcus lactis*	*Listeria monocytogenes Staphylococcus aureus Clostridium tyrobutyricum*	Dal Bello et al., 2010; Biscola et al., 2013; Parapouli et al., 2013
Pediocin	*Pediococcus* spp.	*Listeria monocytogenes Brochothrix* spp.* Clostridium* spp.* Bacillus* spp.* Staphylococcus* spp. *Enterococcus* spp.	Šuškovič et al., 2010; Keşka et al., 2017
Plantaricin	*Lactobacillus plantarum*	*Bacillus cereus Listeria monocytogenes Staphylococcus aureus Brochothrix thermosphacta Escherichia coli Pseudomonas* spp. *Clostridium tyrobutyricum Clostridium perfringens Enterococcus* spp. *Salmonella* spp.	Todorov et al., 2010
Sakacin	*Lactobacillus sakei*	*Listeria monocytogenes Staphylococcus aureus Brochothrix thermosphacta Enterococcus* spp. *Klebsiella* spp.* Escherichia coli Pseudomonas* spp. *Campylobacter* spp.	Urso et al., 2006
Enterolysin A	*Enterococcus faecalis Enterococcus malodoratus*	*Listeria monocytogenes Staphylococcus aureus*	Šuškovič et al., 2010
Lysostaphin	*Staphylococcus simulans*	*Staphylococcus aureus*	Šuškovič et al., 2010

Bacteriocin activity has been reported to be less effective in the products than *in vitro*. This reduction might be due to the binding of the bacteriocin molecules to the food matrix, namely to the fat, but also due to the undermining action of proteases and other enzymes. Furthermore, bacteriocins are unevenly distributed in the food matrix, and may be inhibited by salt and curing agents (Pragalaki et al., 2013). Nevertheless, bacteriocinogenic LAB have been used as bioprotective cultures to prevent the growth of pathogens in sausages. In fact, a *Lb. sakei* strain has been used as starter in fermented sausages with a consequent decrease in the numbers of *L. monocytogenes* (De Martinis and Franco, 1998).

In *in vitro* inoculated meat samples, Castellano et al. (2012) demonstrated the ability of a *Lb. sakei* strain in controlling the growth of *S. aureus* and *L. monocytogenes*, assigning this effect to the production of bacteriocins.

Therefore, LAB strains producing bacteriocins are gaining importance in the production of dry-cured and fermented meat products due to their activity against undesirable microorganisms. Numerous studies have shown that LAB can be used to reduce the population of unfavorable microbiota in dry-cured meat products and are likely to have a commercial application in food preservation as natural food preservatives. Due to their antilisterial activity, bacteriocinogenic strains of LAB and their bacteriocins may be beneficial as preservation agents in dry-cured and fermented products, and can be used as technological alternatives to chemical preservatives, meeting the increased demand for foods with few or even no chemical additives (Keşka et al., 2017).

Two *Lactobacillus curvatus* strains isolated from Italian salami produce two bacteriocins, sakacin P and sakacin X, with activity against *L. monocytogenes*. Moreover, the application of semi-purified bacteriocins to the salami batter caused a reduction in the counts of *L. monocytogenes* of 2 log cfu/g in the final product, thus contributing to improve the safety of these type of meat products (de Souza Barbosa et al., 2015).

Bacteriocins and other antimicrobial peptides are becoming more important with the increased resistance of bacteria to traditional antimicrobials. In some clinical cases, LAB and bacteriocins may be the only therapy, sometimes in combination with low dosages of traditional antimicrobials (Mokoena, 2017). Innovative applications of LAB and bacteriocins are progressively emerging, such as site-specific drug delivery and anti-quorum sensing strategies (Mokoena, 2017). Furthermore, peptide engineering is a new approach to design of antimicrobial peptides eventually more powerful and/or more specific (Rossi et al., 2008; Perez et al., 2014).

### Effect of CNS/Micrococcaceae Starters

Staphylococci also play a role in preserving meat products by synthesizing nitric oxide from arginine via nitric oxide synthase (NOS), which is widely distributed in staphylococci (Sapp et al., 2014). This activity was also observed in other staphylococci used as starter cultures in meat (Ras et al., 2017, 2018a,b).

Reduction of nitrate to nitrite in meat is made by nitrate reductase from staphylococci. *S. carnosus* and *S. xylosus* have a nitrite reductase. For *S. carnosus*, this activity is regulated by nitrate, so when the nitrate concentration becomes limiting, the accumulated nitrite, resulting from the nitrate reductase activity, is imported and reduced to ammonia by its nitrite reductase (Neubauer and Götz, 1996).

Nitrate reductase is often described as being involved in the reduction of nitrate to nitrite, but the reduction of nitrite that leads to the production of NO, independently of respiration, can be due to a molybdenum enzyme, such as nitrate reductase (Maia and Moura, 2015). The conditions required to observe NO synthesis by nitrate reductase result from anaerobic conditions associated with a decrease in nitrate concentration combined with the accumulation of nitrite in the medium (Maia and Moura, 2015).

In cured fermented meat products, NOS may help staphylococcal starter cultures to adapt to an oxidative/nitrosative stress environment (Ras et al., 2018b).

### Effect of Yeasts and Molds as Starters

Yeasts and molds are used less frequently as starter cultures. However, the application of molds and yeasts as surface starter cultures may sometimes contribute to an increased product safety (Bosse et al., 2018). Strains of *D. hansenii*, *Debaryomyces maramus*, *Hyphopichia burtonii*, *Penicillium chrysogenum*, and *Penicillium* sp. have been used as starters in the manufacturing of South European dry-cured hams (Martín et al., 2004, 2006; Rodríguez et al., 2015; Simoncini et al., 2015).

## Chemical Hazards in Fermented Meat Products

Amongst the chemical hazards that constitute a major concern in meat products, the most important are biogenic amines (BAs), nitrosamines, polycyclic aromatic hydrocarbons (PAHs), and mycotoxins, among others.

### Biogenic Amines

Biogenic amines (BA) are nitrogenous compounds derived from amino acids (Suzzi and Torriani, 2015; Elias et al., 2018).

The content and profile of BA present in fermented meat products has been extensively studied (Suzzi, 2003; Roseiro et al., 2010; Laranjo et al., 2016, 2017b).

Histamine, tyramine, and phenylethylamine are the foremost dietary BA associated with health problems, namely vasoactive and psychoactive reactions: histaminic intoxication, enteric histaminosis causing food intolerance, food-induced migraines, and interactions between tyramine and monoamine oxidase inhibitors (Spano et al., 2010; Linares et al., 2011).

The production of fermented meat products involves a hugely diverse microbiota that includes technologically important microorganisms as well as undesired food spoilers and pathogens (Latorre-Moratalla et al., 2012). High levels of BA have traditionally been used as an index of undesired microbial activity in food, which may derive from poor hygiene manufacturing or storage practices (Mariné-Font et al., 1995; Suzzi, 2003; Ruiz-Capillas and Jimenez-Colmenero, 2004). Nevertheless, both technological microbiota as well as microbial contaminants may be responsible for producing BA (Latorre-Moratalla et al., 2012). Therefore, it is necessary to effectively control the levels of BA that accumulate in fermented meat products, due to the health risks associated with these compounds (Latorre-Moratalla et al., 2012; Vidal-Carou et al., 2015).

Some toxicological characteristics and outbreaks of food poisoning are associated with histamine and tyramine (Silla Santos, 1996).

Several studies have demonstrated the role of starter cultures in reducing the accumulation of BA in meat products (Maijala et al., 1995; Hernández-Jover et al., 1997; Bover-Cid et al., 1999; Baka et al., 2011; Lu et al., 2015). Nevertheless, other studies have reported the inefficiency of starters to reduce the content in BA in some fermented meat products (Parente et al., 2001; Bozkurt and Erkmen, 2002). Furthermore, Komprda et al. (2004) have shown that the effect of starter cultures in avoiding the accumulation of BA strongly depends on the strain(s) used.

Recent studies have shown that autochthonous starter cultures may control the accumulation of BA in fermented meat products, while retaining their sensory properties (Lorenzo et al., 2017).

Hernández-Jover et al. (1997) reported a slight reduction in the contents of tyramine and cadaverine throughout the curing of sausages inoculated with two mixed starter cultures, one of *Micrococcus carnosus* plus *Lb. plantarum* and another of *M. carnosus* plus *Pediococcus pentosaceus*.

Other authors found a significant decrease in the levels of tyramine, cadaverine, and histamine during the ripening of sausages with combined staphylococci and lactobacilli starter cultures (Maijala et al., 1995).

Combined starter cultures of *Lb. sakei* with either *S. carnosus* or *S. xylosus* amine-negative strains inoculated in fermented sausages resulted in a severe reduction in the contents of tyramine, cadaverine, and putrescine, when compared with spontaneously fermented sausages (Bover-Cid et al., 2000).

On the contrary, the use of *P. pentosaceus* and *S. xylosus* strains as starter cultures was not able to prevent the accumulation of putrescine and tyramine produced by some indigenous LAB, therefore revealing a non-optimal use of starters (Pasini et al., 2018).

### Nitrosamines

In food microbiology, BA have sometimes been related to spoilage and fermentation processes. These amines can undergo nitrosation to form nitrosamines, mainly in the presence of nitrites (Ruiz-Capillas and Jimenez-Colmenero, 2004). Nitrite can be converted to nitric oxide, a nitrosating agent that can react with amines to produce nitrosamines. In fact, nitric oxide can react with secondary amines to produce potent carcinogenic nitrosamines. These are more stable than those formed from primary amines that break down quickly, whereas tertiary amines can hardly form nitrosamines (Douglass et al., 1978).

According to Reig and Toldrá (2015), the main nitrosamines occurring in meat products are listed in Table 2.

**Table 2:** Main nitrosamines occurring in meat products.

Nitrosamine	Abbreviated name
N-nitrosodimethylamine	NDMA
N-nitrosopyrrolidine	NPYR
N-nitrosopiperidine	NPIP
N-nitrosodiethylamine	NDEA
N-nitrosodi-n-propylamine	NDPA
N-nitrosomorpholine	NMOR
N-nitrosoethylmethylamine	NEMA

**FIGURE 1:**
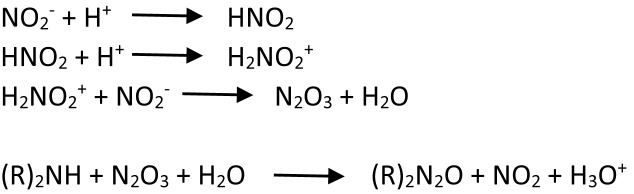
Reduction of nitrite to nitrous anhydride, followed by nitrosation of a biogenic amine with nitrous anhydride.

In foods, nitrosamines are formed by reactions of nitrogen oxide with amines. Initially, nitrite in food, added as a preservative, is hydrogenated to hydronitrogenoxide (H_2_NO_2_^+^) under acidic conditions. This compound reacts with another molecule of nitrite to form nitrogen anhydride after dehydration, which donates nitroso group to the amines in food to produce N-nitrosamines (Rostkowska et al., 1998). The formation of N-nitrosamines is illustrated in Figure 1 adapted from Rostkowska et al. (1998).

The International Agency for Research on Cancer (IARC) of the World Health Organisation (WHO) has reported an association between the consumption of meat and processed meat products and the risk of colorectal cancer (Bouvard et al., 2015; IARC, 2015). Nevertheless, the probability of formation of stable N-nitrosamines in meat and meat products is rather low (Honikel, 2008).

According to Tanaka et al. (1985), the bacon produced in different industries with 80 or 40 ppm of sodium nitrite and a culture of *P. acidilactici* (7.0 log cfu/g bacon) had a significantly lower content in nitrosamines than a bacon commercially produced with the usual formula containing 120 ppm of sodium nitrite.

The biodegradation of nitrite by LAB may occur due to the acidification of the product or to the action of nitrite reductase. Indeed, LAB, mainly lactobacilli and pediococci, significantly contribute to nitrite-depletion in cured meats, which is increased by the decrease in pH due to the lactic acid produced (Dodds and Collins-Thompson, 1984).

In recent years, the use of natural curing agents, such as celery, containing nitrate, combined with nitrate reducing starter cultures, has been proposed to minimize the use of nitrite (Sebranek et al., 2012).

### Polycyclic Aromatic Hydrocarbons (PAHs)

Polycyclic aromatic hydrocarbons (PAHs) are aromatic hydrocarbons with two or more combined benzene rings in different conformations (Lawal, 2017), that do not contain heteroatoms or carry substituents (Wenzl et al., 2006).

PAHs containing up to four rings are referred to as light PAHs, while those containing more than four rings are considered heavy PAHs (Wenzl et al., 2006). Heavy PAHs are more stable and more toxic than the light PAHs (Lawal, 2017).

One of the main source of human exposure is the dietary intake of PAHs (Duan et al., 2016). Food products can be contaminated by PAHs that exist in the surrounding environment, but also throughout food processing and cooking (Lawal, 2017). Smoking is a traditional curing process that is applied to certain type of cured meat products. Smoking serves preservation purposes, since it inhibits the growth of molds and bacteria on the product surface, but also delays lipid oxidation, and adds a characteristic smoky flavor (Holck et al., 2017).

Several PAHs have been considered by the IARC and the European Union due to their carcinogenic and mutagenic properties (EFSA, 2008; Singh et al., 2016), 15 PAHs showing clear evidence of mutagenicity/genotoxicity, and benzo(a)pyrene (BaP) having been reported to be carcinogenic. Thus, particular attention has been given to a group of eight PAHs (PAH8), which were used in previous cancer studies and in EFSA’s risk evaluation (EFSA, 2008). Furthermore, the EFSA concluded that BaP is not a suitable indicator for the occurrence of PAHs in food, since several foods contain PAHs although no BaP was detected. Hence, the EFSA panel proposed the use of a specific group of four-PAH4 (benzo(a)pyrene, benz(a)anthracene, benzo(b)fluoranthene, and chrysene) or eight-PAH8 (benzo[a]pyrene, benz[a]anthracene, benzo[b]fluoranthene, benzo[k]fluoranthene, benzo[ghi]perylene, chrysene, dibenz[a,h]anthracene, and indeno[1,2,3-cd]pyrene) PAHs to evaluate the occurrence and toxicity of PAHs in food products (EFSA, 2008). Therefore, the Commission Regulation (EC) No 835/2011 established an upper limit for PAHs (BaP and PAH4) in smoked meat and smoked meat products (EC, 2011): 2.0 μg/kg for BaP and 12.0 μg/kg for PAH4.

PAHs profiles in smoked meat products have been studied in different product types and manufacturing practices (smoking wood, smoking practices) (Roseiro et al., 2011; Santos et al., 2011; Gomes et al., 2013).

PAHs profiles of Portuguese traditional dry-fermented sausages from south Portugal have been studied (Santos et al., 2011; Gomes et al., 2013). However, the effect of starters on PAHs content has been given little attention. Elias et al. (2014) studied Portuguese traditional sausages and concluded that the use of starter cultures did not affect their content in PAHs.

### Mycotoxins

Mycotoxins are secondary fungal metabolites capable of causing disease that may vary widely in their toxicity. They have a number of adverse effects on health, affecting the immune system, nervous system, liver, kidneys, blood, and some mycotoxins are known to be carcinogens.

The toxic effects of mycotoxins may be either acute (after a single exposure) or chronic (after repeated exposure).

The most important mycotoxins in terms of effects on health are the aflatoxins, ochratoxin A (OTA), patulin and the *Fusarium* toxins. Aflatoxins are considered to be the most toxic ones and long-term low level exposure to aflatoxins has been associated with liver diseases (Afum et al., 2016).

Maximum levels have been set for the major mycotoxins in food by Commission Regulation (EC) No 1881/2006 (EC, 2006), the EU legislation which sets maximum levels for chemical contaminants in foodstuffs, as amended by Commission Regulation (EC) No 1126/2007 (EC, 2007).

Recent studies have highlighted the presence of OTA, an important secondary metabolite of several fungi belonging to the genera *Penicillium* and *Aspergillus*, in dry-cured hams and sausages of different origins (Iacumin et al., 2009; Rodríguez et al., 2012; Comi and Iacumin, 2013; Rodríguez et al., 2015).

The inoculation of a *P. chrysogenum* strain together with selected autochthonous non-toxigenic mold strains on the surface of dry-cured Iberian hams limited the growth of OTA-producing molds, and prevented the accumulation of OTA in dry-cured Iberian hams throughout ripening (Rodríguez et al., 2015). Furthermore, *Candida guilliermondii*, *Endomycopsis fibuliger*, and *P. nalgiovense* isolated from dry-cured hams were able to prevent the growth of mycotoxin producing molds on the surface of San Daniele dry-cured ham (Comi and Iacumin, 2013).

## Competitive Exclusion

The use of starters, particular LAB, as competitive microbiota in fermented meat products, may play a double role of inhibiting or controlling the growth of food pathogens or food spoilage microorganisms, with the consequently increased shelf-life, whilst retaining the sensory properties of the products, namely color, flavor, texture, and nutritional value (Reis et al., 2012).

LAB may have a bioprotective effect against other microorganisms, inhibiting or controlling their growth, either by competing for nutrients and/or by producing bacteriocins or other antagonistic compounds, such as organic acids, hydrogen peroxide, or enzymes (Pragalaki et al., 2013).

Starter cultures engage in competitive exclusion, outcompeting spoilage microorganisms for nutrients and oxygen, and adjusting their performance to the environment through quorum sensing (Young and O’sullivan, 2011).

Furthermore, the growth of LAB in meat products may interfere with the growth of spoilage or pathogenic bacteria by competition for nutrients and living space (adhesion) on the product (Pragalaki et al., 2013; Keşka et al., 2017).

*P. acidilactici* has been shown to inhibit *L. monocytogenes* in fermented Wiener sausages (Degnan et al., 1992).

Additionally, the role of molds, such as *P. nalgiovense*, in the competitive exclusion of undesired filamentous fungi, has also been demonstrated (Fierro et al., 2004). Most of these undesired fungi, belong to the genera *Aspergillus*, *Penicillium*, and *Fusarium*, and produce mycotoxins (Sonjak et al., 2011).

In summary, the direct competition between starter cultures and potential food pathogens by competitive exclusion may be an important mechanism to restrict the growth of undesired microorganisms (Di Gioia et al., 2016).

## Conclusion

Starter cultures are an important tool that contributes to ensure the safety of fermented meat products. Indeed, the microorganisms that constitute starter cultures may inhibit or reduce the growth of spoilage and/or pathogenic populations through mechanisms, such as production of certain metabolites or competitive exclusion. Thus, the use of starter cultures may reduce the need for chemical additives, such as nitrites and nitrates. Furthermore, the lower residual levels of nitrates and nitrites detected in fermented meat products inoculated with starter cultures are due to the ability of starters to metabolize those compounds.

Besides their beneficial effect on safety, which should be the main reason for their use, starters may play other important roles in fermented meat products, such as increasing the reproducibility of product characteristics between batches, shortening the manufacturing times, and improving sensory characteristics.

Although the positive effect of starters in the control or reduction of the microbiological hazards present in fermented meat products, with the concomitant reduction in the levels of biogenic amines, has been extensively studied, more studies are needed on the role of starters in controlling the content in nitrosamines or polycyclic aromatic hydrocarbons.

## Author Contributions

ML drafted the whole manuscript. MP drafted the lactic acid bacteria section of the manuscript. ME critically revised the manuscript and drafted the conclusions. All authors contributed to the writing and the critical revision of the manuscript.

## Conflict of Interest Statement

The authors declare that the research was conducted in the absence of any commercial or financial relationships that could be construed as a potential conflict of interest.
